# Patient perceptions and use of non‐statin lipid lowering therapy among patients with or at risk for atherosclerotic cardiovascular disease: Insights from the PALM registry

**DOI:** 10.1002/clc.23625

**Published:** 2021-05-18

**Authors:** Angela Lowenstern, Shuang Li, Ann Marie Navar, Salim S. Virani, Veronique L. Roger, Jennifer G. Robinson, Anne C. Goldberg, Wendy Kampman, Eric D. Peterson, Tracy Y. Wang

**Affiliations:** ^1^ Duke Clinical Research Institute Durham North Carolina USA; ^2^ Division of Cardiology, Department of Medicine Duke University School of Medicine Durham North Carolina USA; ^3^ Department of Medicine Baylor College of Medicine Houston Texas USA; ^4^ Department of Health Sciences Research and Department of Cardiovascular Diseases Mayo Clinic Rochester Minnesota USA; ^5^ Department of internal medicine University of Iowa Iowa City Iowa USA; ^6^ Department of internal medicine, division of endocrinology Washington University St. Louis Missouri USA; ^7^ Medical affairs Regeneron Pharmaceuticals, Inc. Tarrytown New York USA

**Keywords:** lipid‐lowering therapy, primary prevention, secondary prevention, statin

## Abstract

**Background:**

Non‐statin lipid lowering therapies (LLTs) provide additional treatment options for patients. Use patterns and patient perceptions of non‐statin LLT remain incompletely described.

**Hypothesis:**

The guideline‐recommended statin intensity remains underutilized in patients treated with and without non‐statin LLT.

**Methods:**

The PALM Registry collected LLT information on patients with or at risk of ASCVD treated at 125 US clinics in 2015. We compared patient perceptions, lipid levels and statin use among patients treated with and without non‐statin LLT.

**Results:**

Among 7720 patients, 1930 (25.0%) were treated with a non‐statin LLT (1249 fish oil, 417 fibrates, 329 ezetimibe, 196 niacin). Concurrent statin treatment occurred in 73.7%, of which 45.4% were dosed under the guideline‐recommended intensity. Compared with patients on statin alone, patients receiving both a statin and non‐statin LLT (*n* = 1423) were more likely to be male, white race and to perceive themselves as higher risk of ASCVD compared with their peers (38.5% vs. 34.9%, p = .047). Only 27.4% of patients treated with non‐statin LLT alone perceived themselves at higher risk. Most (75.7%) patients treated with a non‐statin LLT alone reported never being treated with a statin, despite ASCVD in 30.8% of these patients. Among those previously treated with a statin, 59.3% reported being willing to try a statin again.

**Conclusions:**

Non‐statin LLT is used in one in four patients with or at risk for ASCVD; its use is frequently in place of statin therapy or in the absence of guideline‐recommended statin intensity. More work is needed to establish statins as first line therapy.

## 1 BACKGROUND

The benefits of treatment with statins are well recognized among patients with or at risk for clinical atherosclerotic cardiovascular disease (ASCVD). While non‐statin lipid lowering therapies (LLTs), including fish oil, fibrates, ezetimibe and niacin, are also used in clinical practice, randomized trial data have shown mixed results in the ability of these medications to affect clinical outcomes. In the wake of several negative clinical trials, the prescription of non‐statin LLT among Medicare beneficiaries decreased between 2007 and 2011, from 20.5% to 18.9% of patients, driven by a marked reduction in the number of patients prescribed ezetimibe.^1^ Subsequently, in 2013, the American College of Cardiology (ACC)/American Heart Association (AHA) clinical guideline for the management of blood lipid levels established statins as first‐line therapy for patients with or at risk of ASCVD^2^ and recommended consideration of non‐statin LLT in patients who had a response to statin therapy that was less than anticipated or who were unable to tolerate statin treatment. In 2015, the Ezetimibe Added to Statin Therapy after Acute Coronary Syndromes (IMPROVE‐IT) trial showed a clinical benefit in the reduction of cardiovascular events among patients treated with both Ezetimibe and statin therapy as compared with a statin alone.^3^ The 2018 AHA/ACC Multisociety guideline on the management of blood cholesterol also highlighted the need for non‐statin lipid lowering therapy in specific populations of patients deemed to be at high or very high risk of ASCVD events.^4^

Despite the ongoing focus in guideline recommendations on treatment with non‐statin LLT, there remain limited data regarding the treatment patterns, patient perceptions and clinician characteristics associated with non‐statin LLT. Using the Patient and Provider Assessment of Lipid Management (PALM) registry,^5^, ^6^ we aim to: (1) evaluate the characteristics and blood lipid levels of patients treated with non‐statin LLT; (2) examine the clinician characteristics associated with prescribing of non‐statin LLT; and (3) assess the patient perceptions regarding lipid treatment among patients with or without non‐statin LLT.

## 2 METHODS

### 2.1 Study population

The PALM registry is a cross‐sectional registry conducted following the release of the 2013 ACC/AHA lipid guidelines, and which uniquely collected patient and clinician perceptions of ASCVD risk and treatment preferences, as well as detailed patient clinical characteristics and core lab lipid measurements. Between May 27, 2015 and November 12, 2015, the PALM registry prospectively enrolled 7938 patients from 140 outpatient cardiovascular, endocrinology and primary care practices across the United States. Full details regarding the design, rationale, inclusion and exclusion criteria for the PALM registry have been previously published.^5^ In brief, patients were eligible for enrollment if they were treated with a statin or had an indication for statin therapy under the 2013 ACC/AHA guideline. Prior to enrollment, all patients provided signed informed consent. Each site obtained institutional review board approval for participation.

Starting with the overall population of patients, we sequentially excluded patients who did not have completed chart abstraction (*N* = 34), those who did not have core lipid sample results available (*N* = 182) and those missing data on statin use (*N* = 2). This left a final study population of 7720 patients. Among these patients, 7676 (99.4%) had an identified clinician (*N* = 453) who completed a provider survey from 125 clinic sites, allowing for the linkage of patient data with clinician characteristics and survey responses.

### 2.2 Data collection and definitions

At the time of enrollment, medical history, focused on cardiovascular disease history, interventions or risk factors, and sociodemographic information, including race, zip code and insurance payor, were abstracted from each patient's medical record. Current and prior treatment with statin and non‐statin LLT were also recorded. Treatment with PCSK9 inhibitors were not collected as this study was conducted prior to widespread availability. Each patient underwent phlebotomy with total cholesterol, direct low‐density lipoprotein cholesterol (LDL‐C), high‐density lipoprotein cholesterol (HDL‐C) and triglyceride levels measured by a core laboratory at LabCorp (Burlington, NC).

Each patient enrolled in the PALM registry was asked to complete a survey (Table S1) which examined the perception of their own cardiovascular risk relative to their peers, as well as their beliefs about the benefit and risks associated with statin therapies. The survey asked patients to indicate the level to which they agreed or disagreed (strongly agree, agree, neither agree nor disagree, disagree, strongly disagree, do not know/not sure) with statements such as “Statins are safe medications”, “I think statins can cause diabetes” and “Statin medications are effective in reducing the risk of heart disease and stroke”. For patients with current or prior statin or non‐statin LLT use, the survey asked patients to select the reason(s) for taking each medication from a list which included “my ‘bad’ cholesterol was too high”, “to prevent stroke”, “to prevent heart attack”, among others. The survey also queried prior adverse symptoms related to prior or current statin therapy and interventions attempted to reduce symptoms. Questions and answer choices are shown in Table S1.

Clinicians treating the enrolled patients were asked to complete a survey querying their specialty, number of years in practice, and whether their clinical practice was associated with a teaching institution. Clinicians were also asked to indicate how they would respond to a hypothetical patient already on atorvastatin 80 mg daily for secondary prevention who still had suboptimal lipid levels (total cholesterol 220 mg/dl, LDL cholesterol 130 mg/dl, and HDL cholesterol 30 mg/dl); potential answer choices included add ezetimibe, add fibrate, add fish oil, add bile acid sequestrant, change to a different statin, refer to lipid specialist, no change, and other.

### 2.3 Statistical analysis

The proportion of patients treated with each non‐statin LLT (fish oil, fibrate, ezetimibe, niacin) was described. Concomitant statin use, as well as the frequency of guideline recommended statin intensity was summarized based on non‐statin LLT treatment.

We linked each surveyed clinician to the patients they treated in PALM. The clinician characteristics were compared for patients receiving non‐statin LLT treatment versus those who did not. We evaluated the clinician characteristics associated with the choice to add a non‐statin LLT for the hypothetical patient with persistently elevated LDL‐C despite high intensity statin use.

Patients were then grouped according to current LLT including statin alone, statin plus non‐statin LLT, non‐statin LLT alone and neither a statin nor a non‐statin LLT. Between the first two groups, then between the latter two groups, baseline patient demographic and clinical characteristics were compared. Additionally, we assessed patient perceptions of their own cardiovascular risk, beliefs about effectiveness and safety of statin therapy. Among current and prior statin users, we assessed history of symptoms associated with statin therapy, methods used to reduce these symptoms, and willingness to try another statin medication in those who discontinued statin therapy. Categorical variables were presented using percentages and continuous variables were presented using medians (25th and 75th percentiles). The Chi‐Square test was used for all categorical variables and Wilcoxon rank sum test was used to compare differences in medians of continuous variables.

Finally, blood lipid levels and proportion of patients achieving an LDL‐C < 100 mg/dl were compared among patients treated with a statin alone versus those treated with a statin and a non‐statin LLT. In a similar manner, blood lipid levels were examined based on which non‐statin LLT patients were prescribed.

For each analysis, a p value of <.05 was considered significant. All statistical analyses were performed at the Duke Clinical Research Institute using SAS version 9.4 (SAS Institute, Inc., Cary, NC).

This study was supported by Sanofi and Regeneron Pharmaceuticals. The authors are solely responsible for the design and conduct of this study; all study analyses, the drafting and editing of the manuscript and its final contents.

## 3 RESULTS

### 3.1 Non‐statin lipid lowering treatment patterns

Among 7720 patients with or at risk for ASCVD treated at 125 cardiology, primary care, or endocrinology clinics across the US, 1930 (25.0%) were prescribed a non‐statin lipid lowering medication. This included 1249 patients treated with fish oil (64.7%), 417 patients treated with fibrates (21.6%), 329 with ezetimibe (17.0%) and 196 with niacin (10.2%). Among these patients, 278 (14.4%) were treated with more than one non‐statin LLT. Combination therapy with fish oil and a fibrate was most frequently used (29.8%) followed by fish oil and niacin in 24.1% of patients treated with more than one non‐statin LLT.

Physician characteristics associated with each non‐statin LLT use are shown in Table 1. Patients treated with fish oil products were more likely to be seen by cardiologists (41.4% vs. 31.0%, p < .001) or clinicians who practiced at a teaching institution (18.8% vs. 14.9%, p < .001). In contrast, patients treated with fibrates were less likely to be seen by a physician practicing at a teaching institution (9.1% vs. 15.9%, p < .001). Ezetimibe‐treated patients were more likely to have been seen by a clinician with over 10 years in practice (90.7% vs. 84.1%, p = .003) but who was less likely to practice at a teaching institution (6.1% vs. 15.9%, p < .001). Niacin was infrequently used and patients prescribed this medication were more likely to be seen by a cardiologist (51.9% vs. 32.2%, p < .001).

**TABLE 1:** Physician characteristics for patient treatment with each non‐statin LLT

	Fish oil *N* = 1249	No fish oil *N* = 6471	Fibrate *N* = 417	No Fibrate *N* = 7303	Ezetimibe *N* = 329	No Ezetimibe *N* = 7391	Niacin *N* = 196	No Niacin *N* = 7524
Cardiologist	41.4%	31.0%	31.6%	32.8%	33.5%	32.7%	51.9%	32.2%
p value	**<.001**		0.63		0.74		**<.001**	
>10 years in Practice	87.8%	83.7%	88.1%	84.1%	90.7%	84.1%	88.1%	84.2%
p value	**<.001**		.045		**.003**		0.16	
Practices at Training Institution	18.8%	14.9%	9.1%	15.9%	6.1%	15.9%	20.0%	15.4%
p value	**<.001**		**<.001**		**<.001**		0.10	

Bold values are significant (*p* < .05).

When patients treated with a non‐statin LLT were asked the reason for treatment, 61.0% reported that a non‐statin LLT was prescribed because “bad cholesterol level was too high”, 27.3% believed that the non‐statin LLT medication was prescribed to “prevent stroke” and 38.9% believed that the non‐statin LLT was prescribed to “prevent heart attack”. Table 2 shows the patient responses for treatment indication based on non‐statin LLT prescribed.

**TABLE 2:** Patient survey responses for indication of non‐statin LLT

	Non‐statin LLT overall^a^	Fish oil	Fibrate	Ezetimibe	Niacin
Bad cholesterol too high	61.0%	57.8%	69.0%	76.9%	59.0%
Triglyceride too high	39.6%	34.2%	63.5%	43.4%	42.5%
Good cholesterol too high	20.3%	18.3%	25.2%	24.5%	38.1%
Prevent stroke	27.3%	28.7%	21.7%	34.4%	29.9%
Prevent heart attack	38.9%	41.9%	28.6%	42.9%	45.5%
Family history	32.2%	31.7%	34.5%	33.0%	32.8%
Do not know	11.1%	12.4%	6.2%	10.9%	11.9%

a Among patients who could be linked with survey data including: 1187/1249 patients treated with fish oil, 396/417 patients treated with fibrate, 314/329 patients treated with ezetimibe and 186/196 patients treated with niacin.

### 3.2 Concurrent statin therapy

Overall, 1423 (73.7%) patients were concurrently treated with a statin. Patients treated with niacin had the highest proportion of concomitant statin treatment (82.1%) while those treated with fish oil had the lowest proportion of statin treatment (71.7%) and high‐intensity statin use (31.8%, Table 3). When used concurrently, statins were most commonly prescribed at a moderate intensity, and 45.4% of patients were on a statin intensity less than that recommended in the 2013 ACC/AHA cholesterol guideline.

**TABLE 3:** Statin use among patients treated with non‐statin LLT

	Patients with non‐statin LLT overall *N* = 1930	Patients treated with fish oil *N* = 1249	Patients treated with fibrate *N* = 417	Patients treated with ezetimibe *N* = 329	Patients treated with niacin *N* = 196
Statin (overall) at visit	73.7%	71.7%	74.8%	79.9%	82.1%
High intensity^a^	32.1%	31.8%	36.9%	34.4%	35.4%
Moderate intensity^a^	57.9%	57.7%	55.0%	54.8%	60.9%
Low intensity^a^	10.0%	10.5%	8.1%	10.8%	3.7%
Lower than guideline‐recommended statin dose	45.4%	44.4%	46.7%	47.4%	40.0%

^a^ Among patients with a statin at the visit.

In a hypothetical scenario of a patient with ASCVD and suboptimal lipid levels (total 220 mg/dl, LDL‐C 130 mg/dl, and HDL‐C 30 mg/dl) despite adherence to high intensity statin, 47.5% of clinicians reported that they would add a non‐statin LLT (40.2% ezetimibe, 3.8% fish oil, 3.3% fibrate, 0.2% other). Clinicians who chose to add a non‐statin LLT were no more likely to be cardiologists (51.2% vs. 47.4%, p = .449) and to have been in practice for at least 10 years (80.5% vs. 60.9%, p = <.0001), without difference in teaching institution status (21.8% vs. 26.8%, p = .24) as compared with clinicians who elected not to add a non‐statin LLT.

### 3.3 Patient treated with combined statin/non‐statin LLT versus statin alone

Supplemental Table S2 describes patient characteristics in four groups: patients treated with combined statin/non‐statin LLT, patients treated with statin alone, patients treated with non‐statin LLT alone, and patients treated with neither.

Patients treated with a combination of statin plus non‐statin LLT were more frequently male (63.5% vs. 53.7%, p value <.0001), of white race (90.4% vs. 83.6%, p value <.0001), and college or higher educated compared with patients treated with a statin alone (40.6% vs. 34.7%, p value = .0001). These patients were significantly more likely to have had prior ASCVD (57.5% vs. 48.0%, p < .0001) or a family history of premature ASCVD (41.9% vs. 37.0%, p = .001), as well as cardiovascular risk factors such as hypertension (83.1% vs. 80.4%, p value = .02); they were less likely to engage in tobacco use (9.42% vs. 11.9%, p value <.001). Compared with patients on statin alone, patients on combination statin/non‐statin LLT were similarly likely to be on a statin dose lower than recommended by the ACC/AHA guidelines (41.9% vs. 43.8%, p = .26).

When patients treated with a statin and non‐statin LLT were asked the reason for treatment, 62.8% reported that a non‐statin LLT was prescribed because “bad cholesterol level was too high” and 44.9% believed that the non‐statin LLT medication was prescribed to “prevent stroke or heart attack”. Nearly half (45.4%) of the patients reported a history of symptoms related to statin therapy, including 34.7% with prior muscle cramps, and 46.1% of these had previously made changes to statin dose/type to reduce side effects. Among patients on a non‐statin LLT and a statin dosed under the guideline‐recommended intensity, 154 (52.9%) reported a history of symptoms related to statin therapy. Patients on combination statin/non‐statin therapy were more likely to perceive themselves to be at higher risk for a heart attack or stroke compared to their age‐ and gender‐ matched peers (38.5% vs. 34.9%, p = .047) and to agree that statins are effective (80.2% vs. 76.1%, p = .01) than patients treated with statins alone. Similar proportions of patients in each group agreed that statins are safe (58.7% vs. 60.8%, p = .28).

**FIGURE 1:**
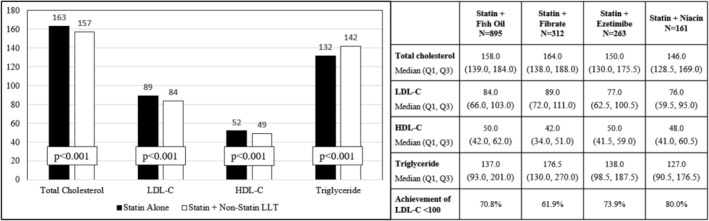
Blood lipid levels among patients treated with a statin or combination statin and non‐statin lipid lowering therapy

Patients treated with combination statin and non‐statin LLT had more favorable lipid profiles, including lower HDL‐C (median 49.0 vs. 52.0 mg/dl, p < .001) levels compared with patients treated with a statin alone (Figure 1). The median LDL‐C was also lower among patients treated with combination therapy (median 84.0 vs. 89.0 mg/dl, p < .001) and a significantly greater proportion of patients on both a statin and non‐statin LLT achieved an LDL‐C < 100 mg/dl (69.5% vs. 63.3%, p < .001). Conversely, median of triglyceride levels were higher among patients treated with both a statin and non‐statin LLT as compared with those on a statin alone (median 142.0 vs. 132.0 mg/dl, p < .001).

### 3.4 Patient treated with LLT alone versus no treatment

Among patients not on statin therapy, those treated with a non‐statin LLT were more likely to be male, of white race, and less likely to be college or higher educated than patients receiving no LLT. They were also more likely to have prior ASCVD (30.8% vs. 22.8%, p < .001) than patients receiving no LLT. The majority of patients (75.7%) in the non‐statin LLT group had never previously attempted taking a statin; however, prior statin therapy was more frequent in this group compared with patients on no LLT (24.3% vs. 18.8%, p = .007). Among those patients on non‐statin LLT who reported previously being treated with statin therapy, 60.3% cited side effects as the reason for stopping statin therapy. However, 22.1% reported “almost certainly” being willing to try another statin and an additional 37.2% reported that they would possibly or very likely be willing to try another statin.

**FIGURE 2:**
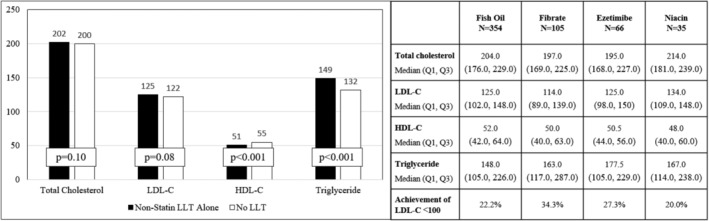
Blood lipid levels among patients treated with non‐statin lipid lowering therapy versus no lipid lowering therapy

While patients on non‐statin LLT alone were no more likely to perceive themselves to be at “slightly higher” or “much worse” risk for a heart attack or stroke compared to their peers (27.4% vs. 27.3%, p = .98), they were more likely to agree that statins are effective (60.7% vs. 49.2%, p < .001) and that statins are safe (38.3% vs. 28.9%, p = .002) when compared with patients treated with neither statin nor non‐statin LLT. The median total cholesterol and LDL‐C values were similar between the groups, although patients without any LLT were observed to have higher HDL‐C and lower triglyceride levels (Figure 2).

## 4 DISCUSSION

In this analysis of the PALM registry, we examined the use of non‐statin LLT as well as the patient and clinician characteristics associated with use of non‐statin LLT. We observed that: (1) one in four patients with or at risk for ASCVD were treated with a non‐statin LLT with fish oil being the most frequently used non‐statin LLT; (2) non‐statin LLT users were frequently not taking statin therapy (25%) or taking a statin intensity less than the guideline‐recommended statin intensity (33%); (3) patients treated with both a statin and a non‐statin LLT had higher clinical risk and rated themselves at higher risk for cardiovascular events than patients treated with statins alone; and (4) patients treated with a non‐statin LLT alone had lower self‐perceived ASCVD risk, and the majority had never received statin therapy. The 2013 ACC/AHA guideline for treatment of blood cholesterol levels recommended statins as the primary lipid lowering strategy with consideration of non‐statin LLT in high‐risk patients who do not have the expected LDL‐C response to statin therapy, who are unable to tolerate a guideline‐recommended statin intensity or who were unable to take a statin due to statin‐associated side effects (SASEs).^2^ These recommendations did not change substantially in the 2018 cholesterol guidelines, other than the addition of PCSK9 inhibitors as a non‐statin LLT option.^4^ While our study preceded FDA approval of PCSK9 inhibitors, the intent was to examine clinician and patient thresholds for the consideration of LLT additions or alternatives beyond first line statin therapy. A quarter of the patients in the PALM registry were treated with a non‐statin LTT. A prior publication from the PALM registry found similar use of non‐statin LLT among patients with a current versus former statin prescription.^7^ Our study builds on this prior analysis by further elucidating the characteristics and beliefs of the patients treated with non‐statin lipid lowering therapy.

Prior literature supports that treatment with non‐statin LLTs can modify lipids.^8^, ^9^ Given its demonstrated LDL‐C lowering and clinical efficacy in secondary prevention patients (IMPROVE‐IT trial), guidelines and expert consensus recommend the addition of ezetimibe as the first non‐statin LLT.^2^, ^4^, ^10^ However, we observed that the majority (64.7%) of non‐statin LLT users were treated with fish oil, a medication that, until the recent REDUCE‐IT trial with optimized doses of icosapent ethyl, did not have strong evidence toward clinical benefit.^11^, ^12^ To date, studies have revealed no clear benefit in the reduction of cardiovascular events with the treatment of fibrates or niacin.^13^, ^14^, ^15^ When patients specified the indication for treatment with a non‐statin LLT, nearly half (45.5%) of patients treated with niacin and 28.6% of those treated with fibrates reported these were to “reduce the risk of heart attack”.

While the rationale for the observed approach cannot be elucidated from the data available in this study, it may be that the majority of patients treated with a non‐statin LLT received fish oil related to its availability as an over‐the‐counter supplement and perceived safety, whereas other non‐statin LLTs require a prescription. Patients treated with fish oil or niacin were more likely to be cared for by a cardiologist; cardiologists were also more likely to add a non‐statin LLT to the patient with elevated blood lipid levels despite the use of high‐intensity statin therapy. This may be related to a greater awareness of the ACC/AHA guideline recommendations for use of non‐statin LLT among clinicians specializing in this field.

Over half of patients receiving both a statin and non‐statin LLT were treated for secondary prevention of ASCVD. This group of patients was more likely to have other high‐risk features, such as diabetes and family history of premature ASCVD. They were also more likely to self‐report higher risk for a cardiovascular event than their age‐ and gender‐matched peers, compared with patients treated with statins alone. Nearly half of the patients reported that the non‐statin LLT was prescribed specifically to prevent cardiovascular events. While there is now randomized clinical trial data to support the benefit of ezetimibe in reducing cardiovascular events,^3^ this was not yet available during the study period. Further, the majority of patients treated with non‐statin therapies received fish oil, niacin or fibrates. In this setting, combination therapy may falsely reassure patients. These results suggest that shared decision‐making conversations should emphasize the significant benefits statins have on cardiovascular outcomes as a first line therapy and the importance of titrating to the maximally tolerated statin dose recommended by the guidelines. Subsequently, in the setting where the LDL‐C level remains suboptimal or when there is intolerance to therapy with the guideline‐directed statin intensity, ezetimibe plays a key role in therapy. This discussion remains relevant in the PCSK9 inhibitor era. PCSK9 inhibitors remain much more cost prohibitive than statin therapy, and over half of patients started on a PCSK9 inhibitor discontinue statin therapy or have an interruption in their PCSK9 inhibitor treatment within a year of initiation.^16^ Thus, statin persistence and dose optimization remain a top priority for patients with or at risk of ASCVD.

The guideline recommendations support statins as the cornerstone of LLT but with the use of ezetimibe as an important adjunct to statin therapy for a patient with known or at high risk for ASCVD who had a response to statin therapy that was less than anticipated, or who were unable to tolerate statin treatment. Unfortunately, non‐statin LLT users in PALM were frequently not on any statin therapy at all, and surprisingly, over 75% of patients treated with a non‐statin LLT alone had never been tried on a statin medication, despite 30% of these having prior ASCVD. Compared with patients on no LLT at all, those treated with non‐statin LLT did not perceive themselves to be at higher ASCVD risk compared with their age‐ and gender‐matched peers, although they were more likely to have established ASCVD, a family history of premature ASCVD, or cardiovascular risk factors. Patients not on statins were less likely to believe statins were effective or safe than patients on statin therapy, but the majority of patients on non‐statin LLT were willing to consider trying a statin. These results suggest that, for these patients, addressing knowledge about own ASCVD risk should be the first step as a lack of awareness of risk likely contributes to under‐treatment. Statin therapy should be introduced as first line therapy after this personalized risk–benefit discussion.

Many non‐statin LLT users, whether current or previous statin users, reported side effects from statin therapy. Prior work from the PALM registry showed that across primary and secondary prevention groups, 55% of patients reported that a statin was discontinued due to SASEs.^7^ Changes to the statin frequency and dose were often attempted to reduce symptoms. Prior findings report 6%–7% of patients experienced some side effects attributed to statin therapy.^17^ However, randomized data has shown no difference in the occurrence of adverse effects, and similarly no difference in discontinuation of therapy, among patients treated with a statin versus placebo.^18^ Guidelines recommend non‐statin LLT use in patients with SASEs or inability to up‐titrate statin dose. While side effects may lead to discontinuation of a statin, readdressing treatment remains a priority. When asked, the majority of non‐statin LLT users who were previously treated with a statin were willing to try treatment with a statin again. The likelihood of statin under‐dosing was similar between patients treated with statin alone versus those treated with combination statin/non‐statin LLT. Statin‐related side effects were present in only a fraction of patients treated with non‐statin LLT and a lower‐than‐guideline‐recommended statin dose. These results suggest that SASEs or inability to up‐titrate the statin was not the primary reason why a non‐statin LLT was added, but even if present, there are opportunities for clinicians to reintroduce statin therapy. Our findings further highlight the gaps in guideline‐directed lipid lowering therapy for patients, including those who have a known history of ASCVD, and the importance of readdressing lipid lowering therapy with open patient discussions at clinical appointments.

### 4.1 Limitations

We acknowledge several limitations associated with our study. First, core laboratory lipid levels were only obtained at the time of study enrollment reflecting lipid treatment received at that time, thus changes in lipids with addition of LLT cannot be described. Second, we were unable to capture motivations behind the lipid‐lowering treatment decisions; some of the patients not treated or under‐treated with a statin based on guideline recommendations may have been decisions made after risk discussions between the clinician and patient. Data for additional non‐statin lipid lower therapies, including bile acids and red yeast rice were collected but there were an insufficient number of patients on these medications to further evaluate in this analysis. For patients using fish oil as a lipid lowering therapy, additional details surrounding this medication – including over the counter versus prescription therapy – were not available. Finally, PCSK‐9 inhibitors were not yet clinically available for use during the enrollment period for the PALM Registry, therefore our description of non‐statin LLTs did not include this class of therapy.

## 5 CONCLUSIONS

One in four patients with or at risk of ASCVD in the PALM Registry was treated with a non‐statin lipid lowering therapy. While ezetimibe is an important adjunct to statin therapy, particularly for those patients without the expected response to a statin or who are intolerant to statins, we found that non‐statin LLT was frequently used in place of statin therapy. Patients treated with a non‐statin LLT alone had lower self‐perceived ASCVD risk, and the majority had never received statin therapy. Further work is needed to address patient knowledge gaps in their own ASCVD risk, as well as to establish statins as first line therapy before considering non‐statin LLT additions or alternatives.

## CONFLICT OF INTEREST

Angela Lowenstern: Dr. Lowenstern reports funding through NIH T‐32 training grant #5 T32 HL069749‐14.

Shuang Li: Ms. Li reports no relevant disclosures.

Ann Marie Navar: Dr. Navar is supported by the NIH, NHLBI K01HL133416–01 and reports research support from Amgen, Sanofi, and Regeneron; consulting fees from Amgen and Sanofi.

Salim S. Virani: Dr. Virani reports research support from ADA/AHA/ VA; honorarium from ACC as the Associate Editor for Innovations, ACC.org.

Veronique L. Roger: Dr. Roger reports no relevant disclosures.

Jennifer G. Robinson: Dr. Robinson reports research support from Amarin, Amgen, Astra‐Zeneca, Eli Lilly, Esai, Glaxo‐Smith Kline, Merck, Pfizer, Regeneron/Sanofi, Takeda; consultant for Amgen, Eli Lilly, Merck, Pfizer, Regeneron/Sanofi.

Anne C. Goldberg: Dr. Goldberg reports research support from Amarin, Amgen, Pfizer, Merck, Regeneron/Sanofi, IONIS, Genzyme/Isis, and Regeneron, Madrigal, and Arisaph; consulting for Optum Rx, Regeneron/Sanofi, and Esperion; honorarium for editorial work Merck Manual.

Wendy Kampman: Dr. Kampman reports employment with Regeneron Pharmaceuticals, Inc.

Eric D. Peterson: Dr. Peterson reports research support from Eli Lilly, Janssen, Merck; Consulting from AstraZeneca, Bayer, Boehringer Ingelheim, Genentech, Janssen, Merck, and Sanofi Aventis.

Tracy Y. Wang: Dr. Wang reports research support from AstraZeneca, Daiichi Sankyo, Eli Lilly, Gilead, Glaxo SmithKline, Regeneron, Sanofi; consultant/advisory/education from Bristol Myers Squibb, Astra Zeneca, Eli Lilly, Premier, Inc.

## Supporting information


**Table S1** Patient Survey Questions and Response Choices

**Table S2** Patient Characteristics Stratified by Lipid Lowering Treatment

## Data Availability

Research data are not shared.
