# Supplementary material for: Patient perceptions and use of non‐statin lipid lowering therapy among patients with or at risk for atherosclerotic cardiovascular disease: Insights from the PALM registry
Source: Clin Cardiol. 2021 May 18;44(6):863–70. doi: 10.1002/clc.23625 (PMC8207979; doi:10.1002/clc.23625)
Supplement: Supplementary file 1 — Table S1 Patient Survey Questions and Response Choices [file CLC-44-863-s001.docx]

| **Table 1. Patient Survey Questions and Response Choices** | |
| --- | --- |
| **Question** | **Response Choices** |
| **Among all patients** | |
| What do you think is your risk of heart attack or stroke compared with other men/women your age? | My risk is much lower than most men/women my age  My risk is slightly lower than most men/women my age  My risk is about the same as most men/women my age  My risk is slightly higher than most men/women my age  My risk is much worse than most men/women my age |
| Statin medications are effective in reducing the risk of heart disease and stroke | Strongly Disagree  Disagree  Neither Agree or Disagree  Agree, Strongly Agree  Don’t Know/Not Sure |
| Statins are safe medications |  |
| **Among non-statin LLT users** | |
| Do you know the reason(s) you are currently taking a [insert non-statin LLT] medication? Please select all that apply. | My “bad” cholesterol was too high  My triglycerides (fats in blood) were too high  My “good” cholesterol was too low  To prevent stroke  To prevent heart attack  Family history of heart disease, stroke or high cholesterol  Other:_____  I am unsure why I am taking this medication |
| **Among current and prior statin users:** | |
| Have you experienced any of the following symptoms while taking a statin? Please select all that apply. | Muscle aches/cramps  Memory loss, forgetfulness, confusion  Weakness  Nausea/vomiting/stomach upset  Constipation  Fatigue  Hives/itching  Other (free text)  I have not experienced any symptoms  I don’t know/can’t remember |
| Did you try any of these methods to reduce or avoid these symptoms? Please elect all that apply. | Reduced the dose  Reduced how often I take it  Temporarily stopped taking it  Switched to another statin  Added another medication to help relieve side effects of statin (e.g. Coenzyme Q10)  Reduced exercise  Other  I didn’t do anything |
| If your doctor recommended it, would you be willing to try another statin to lower your cholesterol or reduce your risk of heart disease? | Not at all  Unlikely  Possibly  Very likely  Almost certainly  I don’t know |
