# Supplementary material for: Patient perceptions and use of non‐statin lipid lowering therapy among patients with or at risk for atherosclerotic cardiovascular disease: Insights from the PALM registry
Source: Clin Cardiol. 2021 May 18;44(6):863–70. doi: 10.1002/clc.23625 (PMC8207979; doi:10.1002/clc.23625)
Supplement: Supplementary file 2 — Table S2 Patient Characteristics Stratified by Lipid Lowering Treatment [file CLC-44-863-s002.docx]

| **Table 2. Patient Characteristics Stratified by Lipid Lowering Treatment** | | | | | |
| --- | --- | --- | --- | --- | --- |
|  | **Overall**  **N=7720** | **Statin Alone**  **N=4096** | **Statin +**  **Non-Statin LLT**  **N=1423** | **LLT Alone**  **N=507** | **Neither**  **N=1694** |
| **Lipid Lowering Therapy** |  |  |  |  |  |
| Statin Intensity |  |  |  |  |  |
| High Intensity | 28.5% | 27.3% | 32.1% |  |  |
| Moderate Intensity | 61.1% | 62.2% | 57.9% |  |  |
| Low Intensity | 10.4% | 10.6% | 10.0% |  |  |
| Non-Statin LLT |  |  |  |  |  |
| Fish Oil | 16.3% |  | 63.3% | 70.1% |  |
| Fibrate | 5.4% |  | 22.4% | 20.9% |  |
| Ezetimibe | 4.3% |  | 18.8% | 13.1% |  |
| Niacin | 2.6% |  | 11.5% | 6.9% |  |
| **Demographics*** |  |  |  |  |  |
| Age, median (Q1, Q3) | 68.0  (59.0, 75.0) | 67.0  (59.0, 75.0) | 68.0  (61.0, 75.0) | 68.0  (58.0, 74.0) | 67.0  (57.0, 74.0) |
| Male sex | 52.6% | 53.7% | 63.5% | 46.1% | 42.7% |
| White Race | 84.8% | 83.6% | 90.4% | 90.3% | 81.2% |
| Insurance |  |  |  |  |  |
| Private | 58.1% | 56.9% | 62.5% | 59.1% | 57.0% |
| Government | 39.6% | 40.9% | 36.6% | 37.9% | 39.5% |
| Other/None | 2.3% | 2.2% | 1.0% | 3.0% | 3.5% |
| College Graduate | 36.1% | 34.7% | 40.6% | 33.1% | 36.5% |
| **Medical History*** |  |  |  |  |  |
| ASCVD | 43.1% | 48.0% | 57.5% | 30.8% | 22.8% |
| Prior MI | 12.6% | 15.1% | 17.1% | 8.3% | 4.1% |
| Heart Failure | 8.7% | 10.0% | 8.9% | 6.4% | 6.0% |
| Hypertension | 77.5% | 80.4% | 83.1% | 72.6% | 67.4% |
| BMI, median (Q1, Q3) | 29.7  (26.1, 34.3) | 29.9  (26.1, 34.5) | 30.1  (26.6, 34.1) | 29.5  (26.3, 34.4) | 29.3  (25.6, 34.0) |
| CKD | 9.5% | 10.0% | 10.8% | 8.7% | 7.4% |
| Diabetes | 38.6% | 41.3% | 43.2% | 31.2% | 30.4% |
| Current smoker | 11.8% | 11.9% | 9.4% | 12.2% | 13.4% |
| Family history of premature ASCVD | 36.3% | 37.0% | 41.9% | 38.9% | 29.1% |
| **Patient Perceptions*** |  |  |  |  |  |
| Higher risk of MI/stroke compared with peers | 33.4% | 34.9% | 38.5% | 27.4% | 27.3% |
| Statins are effective | 70.3% | 76.2% | 80.2% | 60.7% | 49.2% |
| Statins are safe | 52.4% | 60.8% | 58.7% | 38.3% | 28.9% |
| *p<0.01 for all comparisons across the four groups  Continuous variables described as median (25^th^, 75^th^ percentiles), categorical variables described by percentages among non-missing | | | | | |
